# Hospital and Institutional News

**Published:** 1915-01-16

**Authors:** 


### HOSPITAL AND INSTITUTIONAL NEWS.

### THE KING AND QUEEN AT BRIGHTON.

On Saturday last the King and Queen paid a visit to the Indian wounded at the hospital quarters which have been provided for them at the Royal Pavilion and Dome, which was placed at the disposal of the military authorities by the Corporation. Since the buildings, as is well known, have an Oriental atmosphere, there is a certain appropriateness in the surroundings, and several hundred beds are available. Moreover, Saturday was the first fine day at Brighton which the New Year has provided. Sir William Lawrence, administrator of the General Indian Hospital, travelled in the Royal train, which arrived at Brighton at 11.20, and Mr. G. Cooper, chairman of the Brighton Board of Guardians, together with the clerk, Mr. Harris Burfield, were among those who received their Majesties. The Corporation were accorded the King's thanks for their patriotic action, and informed the King, on being asked how the Guardians had provided for the inmates of the Poor-Law institution who had been removed to make room for hospital accommodation, that they had been accommodated in various houses in Brighton. Mrs. Barnato, who has provided a hospital on the front for European officers of the Indian Army, was also presented. The King and Queen were conducted through the wards by Sir W. Lawrence, Colonel Niel Campbell, and Colonel MacLeod. The Dome is fitted up with circular lines of beds, and those patients who were able to be up stood at attention during the inspection. The King talked to many of the wounded, and the Queen, who made many inquiries, showed much sympathy with a soldier who had lost the sight of both eyes. A special visit was also paid to Ganga Singh, a Dogra who has received the V.C. After expressing their approval of the arrangements, the King and Queen inspected York Place Hospital and, after luncheon, the Dyke Road Hospital, where both British and Belgian soldiers are being treated.

### HOSPITAL CHURCH DEDICATED.

In the above connection we may recall that on December 31 the Bishop of Chichester dedicated the temporary church which has been built for the 2nd Eastern General Hospital at Brighton. The building, which adjoins the premises in Dyke Road, has been provided by public subscription through the instrumentality of the Vicar of Brighton, the honorary chaplain, and has been furnished by many gifts and loans. It is lighted by electricity, suitably heated, and has accommodation for about 200 persons.

### THE MASTER OF THE ROTUNDA HOSPITAL'S RESIGNATION.

Dr. Henry Jellett, M.D., F.R.C.P., resident master of the Rotunda Hospital, Dublin, has thrown up his post to join the Army under circumstances that have created considerable discussion. A little while ago Dr. Jellett applied for six months' leave, in order to serve with the Army at the Front. His request, however, was refused by the governors of the hospital, and as soon as he received their refusal Dr. Jellett sent in his resignation and left Dublin. He is understood to be at, or on his way to, the Front, where his intention is to engage in ambulance work. The governors have accepted his resignation, and his work is being carried on by three *locum tenentes* until a new appointment is made. Local medical interest has been aroused by these events, and the opinion has been expressed that the governors might have treated Dr. Jellett more sympathetically. He has held the post since 1911, and, apart from valuable services, has taken a prominent part in the professional life of the city. While it is true that in self-defence hospital committees in certain London and provincial institutions have been compelled to protect their patients' interests by guarding against the complete dislocation of their staffs, we believe that circumstances have rendered this difficulty less pressing in Ireland, as seems evident by the fact that substitutes were at once obtained for the post that the master vacated.

### THE SPECIAL HOSPITAL FOR OFFICERS.

As the first patient arrived last Saturday at the Special Hospital for Officers (suffering from shock), which, thanks to the success that has attended Viscount Knutsford's appeal, has been opened at 10 Palace Green, Kensington, a few words as to the special accommodation offered are of interest. The house, which has been lent by the executors of the late Lord Rendel, is one of the detached houses which overlook Kensington Palace, and enjoy the quiet of the secluded thoroughfare which runs from Notting Hill to Kensington High Street. Accommodation is given to thirty-five patients in separate rooms, except in one or two larger ones in which two beds have been placed. To give the greatest separate accommodation possible partition walls have been placed in some of the ground floor reception rooms, for instance, in that which was formerly the library. The former kitchens are in use; the house possesses a lift, and except for the medical officer's, matron's, and nurses' quarters, consists entirely of bedrooms. There are two bathrooms on the first and second floors and one on the third. Patients are received through the War Office, which has asked those responsible for the hospital to restrict the institution to officers, as the War Office itself is providing a hospital for similar cases among the men. The staff consists of a resident medical officer, Dr. Bousfield, the matron, three trained nurses, and three probationers. It will be remembered that an appeal was issued for £10,000, of which £7,700 has been subscribed. If the remaining £2,300 be subscribed it will be possible to carry on the institution for two years. The house has been furnished by gifts of money and equipment, and, as the requirements are relatively simple, it has been possible to provide a series of quiet, restful bedrooms, of fair size, with the necessary accessories. The rooms are well lighted, and a glimpse of trees, soon to be in leaf, is obtainable through most of the windows. Isolation and quiet on a more complete scale than is possible in ordinary institutions are thus obtainable in this adapted private house.

### A VIGOROUS POLICY FOR HERTFORD COUNTY HOSPITAL.

A special meeting of the governors of Hertford County Hospital was held on Christmas Eve to consider the desirability of selling certain invested funds, to the amount of £1,600, in order to pay off an accumulated overdraft at the bank. After an interesting discussion it was decided, by four votes to three, to give power to the trustees to sell the stock. As will be seen, though the meeting was small, the voting was very close, and the chairman, Mr. A. H. Smith, failed to convince Mr. Clinton Baker and Admiral Hastings of the advisability of the above proceeding which he advocated. Both the latter gentlemen were in favour of an effort being made to raise funds by appealing to the public, on the ground that were no such effort made the financial position of the hospital would be only temporarily alleviated, and that at the end of the year the deficit would almost certainly begin to grow again. The children's ward has been closed already, and, therefore, it is apparent that a vigorous policy is indeed called for. So convinced were Admiral Hastings and Mr. Clinton Baker that a genuine effort to attract new subscribers, and possibly to modify certain existing privileges, would be successful, that, on being challenged by the chairman, Mr. Baker undertook to aid in collecting funds in the county to pay off the debt. He was added to the finance committee.

### HOW TO ENSURE THE APPEAL'S SUCCESS.

The matter is worth emphasising, for the annual meeting is being held this month, and consequently an occasion is afforded for focussing the interest of the county. We believe that Mr. Clinton Baker and Admiral Hastings are striking the right note; and if they are backed up, their spirit has only to be communicated to the county to secure the success of an appeal. It is perhaps too late to secure an expert hospital administrator of recognised standing to address the annual meeting; but that, if possible, should be done to ensure a proper attendance and to arouse the county to the urgency of the matter. Failing this, we believe that a vigorous policy should be followed, and that no mere effort to increase subscriptions should be made without a definite, tangible object in view. The best one, in our opinion, would be the raising of sufficient funds to open the closed ward. Closed wards are always bad policy. No public can take an interest in an institution of which every department is not in full and active working. We congratulate Admiral Hastings and Mr. Clinton Baker on their staunch attitude, and urge them to continue their efforts by starting at once a vigorous appeal, backed by public meetings, and, if necessary, a county district worker's hospital fund on the lines of that now flourishing at Cambridge.

### AMBULANCE CONSTRUCTION.

Only recently we drew attention, in an article contributed by an Officer of the Royal Army Medical Corps at the Front, to the success of the motor ambulance. An attempt is now being made to standardise the type, and a commission has been formed, at the instance of Mr. Henry S. Wellcome, of which Sir Frederick Treves has consented to be chairman. The members include representatives of the heads of the Army and Navy Medical Departments, St. John Ambulance Association, and certain mechanical engineering experts. The Commission will act as a judging committee for the award of the prizes, amounting to £2,000, which the Wellcome Bureau of Scientific Research is to provide. The prizes are offered for the best designs of a body for a field ambulance to fit a standard pattern motor-chassis. Designs must be sent in on or before June 30 next, and details of conditions may be obtained from the Secretary, 10 Henrietta Street, Cavendish Square, W.

### THE EDUCATION OF DEFECTIVE CHILDREN.

The Heritage Schools of Arts and Crafts for crippled boys and girls, Chailey, Sussex, is doing a very useful work in connection with the Guild of Brave Poor Things and the Guild of Play, which is very attractively reflected in a recent elaborate Report which has been issued. These schools are recognised and inspected by the Board of Education, the Home Office, and the London County Council, and their work in enabling crippled children to learn a craft which may make them a livelihood later on is one which carries its own commendation with it. Arising out of the two Guilds in London above mentioned, the Heritage Craft Schools have proved so successful that some twenty affiliated branches exist in various parts of the country. The importance of after care and control, which alone can guarantee the success of the education of the physically or mentally defective child, is realised at Chailey; and one of the most signal examples of the adequacy of the methods employed is that Kitchener's Army contains one of its late scholars. The institution at Chailey is badly in need of support, and now that the Mental Deficiency Act is fixing attention on the problem of one class of defective, the good work of construction already done should not fail of public encouragement.

### A HOUSE SURGEON'S CONFESSION IN A CORONER'S COURT.

The old grievance caused by the non-payment of fees to hospital medical witnesses in Coroners' Courts was illustrated by a remarkable confession from Dr. Claud Hamilton Gould, house surgeon at Guy's Hospital, in the Southwark Coroner's Court recently. After giving evidence he said: "When I was twenty-one I had about £2,000, which I invested in medicine. I am now practically penniless. I was down here at three o'clock, and now it is five minutes to five. By coming here I have lost five guineas, for here I don't get a penny." Dr. Waldo, the Coroner, after expressing his sympathy with the medical witness, pointed out that he had no power to grant a fee, although medical witnesses were paid in all other Courts except the Coroner's. This long-standing grievance, so often referred to in our columns, must be emphasised in season and out of season until that remedy is given which was officially advised seven years ago. The Departmental Committee on Coroners which sat in 1908, and before which, by the way, Dr. Waldo gave evidence to the same effect as his remarks on the present occasion, recommended in its Report that all medical men should be paid for evidence in Coroners' Courts. Royal Commissions have the function of recording angels; they leave the reckoning to posterity. But a valuable sidelight is thrown on the present injustice in the house surgeon's confession of the expenses of medicine, and the actual loss which may result from such service in the public interest as the giving of medical evidence in Court undoubtedly is.

### INSURANCE AGAINST DAMAGE BY HOSTILE AIRCRAFT.

Insurance against risk of damage to hospital buildings by bombs from hostile aircraft is undoubtedly a new question to be considered by hospital managers. Some hospital committees have already covered themselves, and in the case of one of the Kentish hospitals, over which the hostile airman flew in the running fight down the Thames estuary on Christmas morning, the Committee seized the opportunity of covering the whole of the hospital buildings three months ago, when rates at Lloyd's were reasonably low.

### THE FLORENCE NIGHTINGALE STATUE.

The transformation of Waterloo Place, to make room for the two statues of Florence Nightingale and Sidney Herbert on the south side of the Guards' Memorial, is nearing completion. The first step was to move back the memorial 40 feet towards Piccadilly Circus, so as to leave room for the two new statues on part of its site facing Pall Mall. The whole forms a triangle, with the two statues as its base—Florence Nightingale's being at the western and Sidney Herbert's, when moved from its present site in the War Office quadrangle, at the eastern angle. Until the whole is completed and unveiled it is impossible to judge of the total effect, but anything which will detract from the nullity of the Crimean monument and give a grace to Waterloo Place is to be welcomed. At all events the appropriateness of uniting the names of Florence Nightingale and Sidney Herbert with the spot in London associated with the Crimean War cannot be questioned. The sculptor of the Nightingale statue is, of course, Mr. A. G. Walker, who received the commission in May 1912, when a committee was formed under the chairmanship of the late Lord Pembroke. The statue, which is 9 feet in height, and stands on a 10-foot pedestal of red and grey granite, depicts Miss Nightingale lamp in hand, and holding up with her left hand part of the skirt of the period, as if actually on her night round. The face, with its characteristic sweetness of expression and light of intelligence and will, is full of life. On the pedestal are four panels, showing Miss Nightingale with another nurse as the centre of a group of convalescent soldiers; with a medical officer inspecting some cots occupied by wounded; and, thirdly and most appropriately, as an old lady addressing a company of nurses. The fourth panel is simply inscribed "Florence Nightingale, 1820-1910." There is no statue which could be more appropriately unveiled at the present time; and Waterloo Place will take on a new significance by its presence.

### LIMITED COMPANIES AS HOSPITAL SUBSCRIBERS.

Our readers who have from time to time learned of the work performed by Sir Edward Wood in the reconstruction of the Leicester Royal Infirmary will be interested to note that in the balance-sheet of the firm of Freeman, Hardy, and Willis, Limited, of which Sir Edward Wood was so long chairman, a recommendation is made for a grant of £2,000 to local and other charities. This firm has thus marked increased prosperity by larger charitable grants—an example which we hope will be widely followed. It is often urged, and with some reason, that limited liability companies are conscienceless, and are not concerned in works of charity; but that is not universally true, and the best of the greater companies anyway have too much organisation on behalf of their employees to carry on to be oblivious entirely of the need for hospitals.

### CONSIDERATION FOR PATIENTS' RELATIVES.

This question, the practical bearings of which an inculcated tact on the part of every member of the staff can alone solve practically, has received a topical public interest from the suggestion of a juryman at the inquest on the victims of the recent train collision at Ilford, that the brother of the deceased Mr. Allen was not allowed to see the patient on the night of his death. As a reflection was thus cast upon the London Hospital, to which the patient was removed, Mr. E. W. Morris, the secretary, has sent to the papers a report, signed by the sister of the ward in which the patient was. From this it appeared that, the patient becoming worse at 8 p.m., the sister learned that Mrs. Allen and a brother were waiting, to whom she sent a message saying that she would come as soon as possible. She then sent a note to one of the house surgeons, and, after carrying out his instructions, went to Mrs. Allen, from whom she learned that the brother had left but would return. Mrs. Allen then entered the ward and remained with her husband, the sister writes, from 9 p.m. till his death just before midnight. Mrs. Allen, it appears, was then placed in a women's ward, where she remained until the brother arrived an hour later. Mrs. Allen had seen her husband three times previously, and other friends were apparently satisfied with the assurance given to them that perfect quiet was essential to his chance of recovery. We mention these details as showing how everything in these cases depends upon the way in which rules are carried out, and it is part of the training given in a great institution to make tact a habit of mind, for, as a recent article on "Appeals" in The Hospital made quite plain, not only the peace of mind of relatives, but public confidence, and therefore public generosity, depend upon it.

### "WHERE ARE THE NINE?"

Mr. Richard Kershaw, secretary of the Central London Throat and Ear Hospital, has kindly forwarded for our inspection a patient's letter of gratitude, which is worth notice. "As near as I can remember," writes the patient, who, formerly in the Postal Service, is now a private in the Middlesex Regiment, "it is now the anniversary of my admission to your hospital for a mastoid operation, so I take the opportunity of again thanking . . . and all the members of the staff who were so kind to me whilst I was an in-patient. I enclose 5s. postal order towards the extension of your hospital, which is, I believe, projected." "Such letters," Mr. Kershaw remarks, "are not so frequent that we pass them unnoticed," and his is not the only institution which may repeat in modern words the old apostrophe to the cured lepers, only one of whom returned afterwards to express his thanks.

### A MEDICAL SUPERINTENDENT'S SALARY.

The Camberwell Board of Guardians are going to make another attempt to induce the Local Government Board to grant Dr. Keats, the medical superintendent, an extra £50 a year. The authorities at Whitehall, as recorded in The Hospital on each occasion, have twice declined to sanction this additional £50, having some time ago granted him £50, although the Guardians were desirous of showing their appreciation of Dr. Keats' services by giving him the larger sum. They have found that several other medical superintendents in the Metropolis are provided with rations in addition to a house, and they have now decided to ask the Local Government Board to sanction rations being assigned to the medical superintendent to the value of £50. It is to be hoped that this final effort to induce the Local Government Board to do justice to Dr. Keats will be more successful than the previous ones.

### SCULCOATES GUARDIANS' TEMPORARY EXPEDIENT.

The house committee of the Sculcoates Board of Guardians, owing to the difficulty experienced in securing medical assistance, have recommended that Dr. H. Robinson be appointed sole medical officer for the workhouse infirmary for three months at a salary of £400 per annum. This was opposed by certain members of the board, who declared that in normal times Dr. Robinson had expressed himself unable to cope with the whole of the work, and that as the times were abnormal and their efforts to secure an assistant medical officer had failed, other means should be tried to tide them over the difficulty, with due regard to the interests of the patients. Other members pointed out that it was a temporary measure only, and that the medical officer had made himself responsible for seeing that the work was done, and by doing so had got the board out of a difficulty. Opposition to the proposal was eventually overcome. In point of fact the efficiency of this work, about which worthy anxiety was expressed by some members, largely depends upon the medical officer's own interest in it, and though understaffing in every branch has been the classic shortcoming of Poor-Law institutional management, and is never therefore to be winked at, the largely chronic and similar cases which find their way into the sick wards of workhouses may in an emergency be looked after without an assistant, provided that the medical officer really undertakes an additional amount of work. The work of such officers in normal times often leads them to demand assistants, and therefore a special sacrifice at a critical period must not be allowed to become a precedent for the future, either in the interests of the patients or of the medical men.

### THE RED CROSS IN CORNWALL.

The activities of the British Red Cross Society were exemplified in the West by the opening on Wednesday, January 6, of an auxiliary home hospital at Launceston, Cornwall. This hospital has been established in the Town Hall, and suitable provision has been made for about twenty cases. On the day of the inauguration five convalescent cases were received by Mrs. W. F. Thompson, the commandant of the detachment. A short service was conducted by the Rev. Canon Lewis, and the Red Cross flag was hoisted by the organising secretary, Mrs. J. C. Williams. The flag itself was presented to the hospital by Capt. L. Ching, R.N.

### REGISTRATION OF BELGIAN MEDICAL PRACTITIONERS.

In the *London Gazette* of January 8 was published an Order in Council declaring that the second part of the Medical Act, 1886, shall be deemed to apply to the Kingdom of Belgium until it is otherwise ordered. In other words, the Registrar of the General Medical Council is authorised to register as a foreign practitioner in the Medical Register a fully qualified Belgian practitioner who applies for registration and produces the necessary evidence. The Order in Council sets forth that "the Kingdom of Belgium affords, in His Majesty's opinion, to the registered medical practitioners of the United Kingdom such privileges of practising in Belgium as to His Majesty seems just, during the continuance of the present war." The action of the Privy Council will not come as a surprise to those who read the Report of the Departmental Committee on the employment of Belgian refugees. That Report stated, with regard to the medical profession, that it had been suggested that qualified Belgian doctors should be allowed to practise among the refugees, or should work among the wounded Belgian soldiers in this country, and intimated that such practice could be regularised by the extension to Belgium of the provisions to which the Order in Council refers. The Report further stated that the Committee had been informed by the Lord President of the Council that the Belgian Government was prepared at once to issue the decree necessary to enable this course to be adopted, and thus place Belgian doctors possessing a recognised diploma on the same footing as British practitioners.

### THE NEW ULSTER VOLUNTEER FORCE HOSPITAL.

Lady Carson on Friday last week opened the Ulster Volunteer Force Hospital, which by permission of the Belfast City Council has been installed in the Exhibition Hall, while an annexe built in the grounds of Queen's University gives a combined total of some eighty beds. Both the Queen's University authorities, who have given the use of their pathological laboratory and $x$-ray installation, and the committee of the Samaritan Hospital, who have arranged for twenty patients and some nurses to be accommodated, have combined to assist the new institution. In the Exhibition Hall a theatre, furnished by Professor Sinclair, has been provided, while the annexe includes kitchen offices, lavatory accommodation, and heating apparatus. Mr. R. I. Colwell, C.E., has acted as honorary architect, and Miss Bruce has been appointed matron-superintendent. The building has been inspected and approved by a representative of the War Office. The committee of the Samaritan Hospital having placed its beds at the disposal of the Ulster Volunteer Force, bring the total accommodation up to 100, but it is interesting to note that the Samaritan Hospital has been previously a hospital for diseases of women.

### THE SCIENTIFIC PRESS DIARIES.

Two of the most useful, as they are among the most popular of The Scientific Press publications, are the "*Nursing Mirror* Pocket Encyclopædia and Diary" and a companion volume, "The Midwives' Pocket Encyclopædia and Diary." Price sixpence each, they contain more information than any but a professional nurse or midwife could make use of in a life-time, while the diary pages are designed, equally for professional purposes, to give room for the recording of engagements, appointments, and the like. It is an interesting fact that, while idle persons are fond of large diaries to record the number of things which have "happened" to them during the day or week past, busy members of institutional staffs employ them rather for recording future obligations and appointments. Certainly the compilers of these two pocket volumes have been very well served by the publishers. The information contained in them is really useful, comprising just those practical points many of which are meant for works of reference rather than for memorising, and which it is convenient to have in such a handy and attractive form. We are not sure that even other members of the staff might not find them useful.

### THE STAY OF THE WOUNDED IN HOSPITAL.

A side of the work which has been perhaps less insisted on in regard to the voluntary hospitals' work for the wounded is that involved by the prolonged stay in the wards which certain patients require. Thus at the Norfolk and Norwich Hospital, to give an example, there are six patients who were received on November 1, and eight who arrived on November 9. This fact lends additional interest and importance to the new emergency ward which, it is hoped, will be ready for the reception of patients by February 1. The beds thus provided, which can be used for such patients as the above, will thus extend the accommodation of the hospital, and the increased space should, it is thought, enable a large convoy, if necessary, to be received each week. No doubt the most serious cases are in the main dealt with by the military hospitals, but nevertheless the voluntary institutions have their share, which is a matter that is, through the lapse of time, beginning to demand attention.

### A HOSPITAL THE VICTIM OF A DISPUTE.

An unfortunate state of friction has arisen between the Loughborough Hospital and the local workpeople's contribution fund, although the hospital is rather a sufferer from than a party to the dispute. The real disputants are the medical men of the town and the Education Committee, and the allegation was that several recommendations had been refused by the medical men on the ground that the Education Committee should pay for their treatment. After prolonged discussion it was determined to send a resolution to the committee of the hospital stating that unless some guarantee could be given that recommendations when presented would be "honoured," the workpeople would have to consider whether to withhold their subscriptions. Now the phrasing of such a resolution seems to us extremely unwise from the point of view of the workpeople, since in practice it is impossible to guarantee the admission of every case which is presented. The very nature of "recommends" should suffice to show this: they are recommendations, as their name shows; they are not admission orders. Indeed, it seems to us most unfortunate that the hospital should have been made the centre of contention in a dispute which rightly concerns the medical men and the Education Committee. Neither party should allow the hospital to suffer from their differences, and an early conference should be arranged, at which, if public spirit is shown on both sides, a settlement should be perfectly possible. The workpeople should realise that the hospital in season and out of season does a great work for them, which should not be interrupted because the local profession and the Education Committee have differences not primarily of hospital importance at all.

### DOCTORS' GIFT TO A MILITARY HOSPITAL.

The medical men of Newcastle presented on Friday last week a motor-ambulance van, purchased by them, to the 1st Northern General Hospital for service until the end of the war. The presentation, which took place at the Royal Victoria Infirmary, was made by Professor Rutherford Morison, who stated the donors hoped that after a career of usefulness in war-time the ambulance would be returned in as good condition as possible, because they wished it to be used for the civil population. Colonel Gowan accepted the ambulance on behalf of the Northern General Hospital, and said they would see it on the roads at all hours, for calls were frequent, and the need for an ambulance great. He hoped that at the end of the war it would be little the worse for wear. This fine gift from the medical men of Newcastle should prove of first-rate service, and those who need a first-hand tribute to the value under the severest tests of the motor-ambulance to the wounded have only to turn to the striking personal experiences given by an Army doctor at the Front in the article which appears on page 351.

### TWO DOCTORS POISONED.

A curious case of aconite poisoning is reported from Co. Clare, by which one medical practitioner lost his life and another narrowly escaped doing so. It appears that Dr. Paul Ryan, medical officer of Ballina, Co. Clare, being unwell, was visited last Saturday by Dr. John Burke, of Killaloe, but previous to the visit someone else had poured aconite from a broken bottle into another which was labelled "Whiskey." Not being aware of this, the doctors drank part of the contents and immediately became ill. Dr. Burke collapsed in the street on his way home, and died in the afternoon. Dr. Ryan has been reported out of danger. An inquest was opened, but was eventually adjourned till January 22, when it is hoped that the surviving doctor will be able to be present. Pending the result of the inquiry we make no comment on what appears to be one of the most curious instances of poisoning in recent years.

### FROM NURSE TO GENERAL PRACTITIONER.

Two professions are concerned at the death of Dr. Ethel Frances Lamport, L.S.A. Lond., M.D. Brux., who died very suddenly on December 27, 1914, at Well, Long Sutton, Hants, where she had been living since her health obliged her to give up her practice in Bow Road, E. "Dr. Lamport," a friend writes, "took a wide view of all movements connected with the welfare of women and children, and her unobtrusive and incessant work endeared her to hundreds of poor workers, whilst her professional skill entitled her to a leading place amongst general practitioners. For ten years she was public vaccinator for Millwall. She was medical officer to the girls' club connected with Messrs. E. Cook's works. Before taking up medicine Miss Lamport went through full training as a nurse at the London Hospital, and was subsequently made a ward sister. She was always proud to remember that she was one of the first set of probationers introduced there by Miss Lückes, and often spoke of the value of her ward training as a preparation for the medical education which she took up later." There can be few instances of a woman who begins as a nurse and qualifies later as a medical practitioner, and the double insight thus received into the care and attendance of the sick might well be valuable, although the qualities which make a good doctor are not quite the same as those which make a good nurse, as both professions will recognise with a proper pride.

### ANTI-TYPHOID INOCULATION IN AMERICA.

The experiences of the New York Health Department in typhoid immunisation are recorded in a lengthy paper by Dr. L. J. Harris, assistant director of the Bureau of Infectious Diseases, which is published in the official organ of the American Medical Association. The general conclusion drawn from these experiences is that the accurate observations recorded in thousands of cases leave no doubt as to the preventive powers of anti-typhoid vaccination in all but a relatively insignificant few, and that in those subsequently affected it strikingly decreases the morbidity and the mortality. Severe reactions, in the experiences of the Bureau, are comparatively rare and have never left a permanent injury. For a period of at least two years, and possibly more, immunisation is as effective in protecting from an attack of typhoid fever as is a previous attack of the disease itself. The following rules for the avoidance of severe reactions are laid down by the assistant director of the Bureau:—(1) Never administer the anti-toxin to any but the healthy. (2) To permit of slow absorption, avoid puncture of a vein or intramuscular injection. (3) Clean the syringe and sterilise the area for injection, using tincture of iodine for the latter purpose. (4) Allow no hard work or indulgence in alcohol after the injection. (5) Avoid re-injecting in indurated areas. Apropos of this subject it is interesting to note that the French Minister of Marine recently addressed to naval officers a circular-letter making anti-typhoid inoculation obligatory in the Navy, as it already was in the Army.

### THIS WEEK'S DRUG MARKET.

Business has begun to improve and the drug market is quite as active as is usual at this time of the year; in fact, in some departments there is more activity than at normal times. Price changes have not been numerous. Supplies of salicylates are becoming scarcer. Cod-liver oil is in much the same position as it was a week ago, and the same may be said of opium. Morphine and codeine continue to be in good demand. Cantharides are dearer. Diethyl-barbituric acid, for which the short Pharmacopœial name is barbitone, has been advanced in price. Bromides are practically unchanged. Castor oil is dearer. Cream of tartar has rather a lower tendency in price. The price of ergot of rye is well maintained. High prices continue to rule for ipecacuanha. Potassium chlorate is dearer.

### TO OUR READERS.

Contributions are specially invited from any of our readers to these columns. They should deal with topical subjects and news. They must be authenticated for the information of the Editor only. The minimum payment if published is 5s. There is no hard-and-fast rule as to space, but notes of about twenty lines in length are preferred.

